# Global Cerebral Ischemia in Male Long Evans Rats Impairs Dopaminergic/ΔFosB Signalling in the Mesocorticolimbic Pathway Without Altering Delay Discounting Rates

**DOI:** 10.3389/fnbeh.2021.770374

**Published:** 2022-01-04

**Authors:** Alexandre Morin, Marilou Poitras, Hélène Plamondon

**Affiliations:** Cerebro-Vascular Accidents and Behavioural Recovery Laboratory, Behavioural Neuroscience Group, School of Psychology, University of Ottawa, Ottawa, ON, Canada

**Keywords:** global cerebral ischemia, delay discounting, impulsive choice, behavioural impulsivity, predator odour, mesocorticolimbic system

## Abstract

Global cerebral ischemia (GCI) in rats has been shown to promote exploration of anxiogenic zones of the Elevated-Plus Maze (EPM) and Open Field Test (OFT). This study investigated changes in impulsive choice and/or defensive responses as possible contributors of heightened anxiogenic exploration observed after ischemia. Impulsivity was assessed using delay discounting (DD) paradigms, while the Predator Odour Test (PO) served to assess changes in defensive responses towards a naturally aversive stimulus. Male Long Evans rats underwent 9 days of autoshaping training and 24 days of DD training prior to GCI or sham surgery (*n* = 9/group). Post-surgery, rats completed the OFT, EPM, and PO, followed by 6 days of DD sessions. Blood droplets served to evaluate corticosterone secretion associated with PO exposure. With impulsivity being regulated through mesocorticolimbic monoaminergic pathways, we also characterised post-ischemic changes in the expression of dopamine D_2_ receptors (DRD_2_), dopamine transporters (DAT), and 1FosB in the basolateral amygdala (BLA), nucleus accumbens core (NAcC) and shell (NAcS), and ventromedial prefrontal cortex (vmPFC) using immunohistofluorescence. Our findings revealed no impact of GCI on delay discounting rates, while PO approach behaviours were minimally affected. Nonetheless, GCI significantly reduced DRD_2_ and ΔFosB-ir in the NAcS and NAcC, respectively, while DAT-ir was diminished in both NAc subregions. Collectively, our findings refine the understanding of cognitive-behavioural and biochemical responses following stroke or cardiac arrest. They support significant alterations to the dopaminergic mesocorticolimbic pathway after ischemia, which are not associated with altered impulsive choice in a DD task but may influence locomotor exploration of the OFT and EPM.

## Introduction

Global cerebral ischemia (GCI) in rats, which mimics cardiac arrest in humans, is associated with vast neurological damage, notably in the selectively vulnerable hippocampus, and leads to profound functional impairments (Neumann et al., 2013). Interestingly, research has reported GCI rats to show behavioural responses that could indicate a rise in impulsivity and/or behavioural disinhibition after infarct, although these remained formally uncharacterised. Notably, in the Elevated-Plus Maze (EPM), ischemic rats show increased open arm exploration and reduced risk assessment behaviours (Plamondon and Khan, 2005; Yan et al., 2007; Knowles et al., 2016; Lu et al., 2016; Morin et al., 2021). Such responses, commonly interpreted as reduced anxiety, appear atypical considering the post-ischemic dysregulation of the hypothalamo-pituitary-adrenal axis, which results in elevated stress-induced corticotropin-releasing hormone and corticosterone (CORT) release, in part due to hippocampal injury affecting negative feedback mechanisms (Barra de la Tremblaye and Plamondon, 2016). In this context, it is unclear if increased exploration of anxiogenic novel environments in ischemic rodents indicates reduced fear, failure to properly appraise aversive stimuli, and/or behavioural impulsivity (as a form of disinhibition). In fact, Keijzer et al. (2020) recently reported a third of cardiac arrest (CA) survivors to experience attention and awareness deficits, 55% of them showing disinhibition and impulsive behaviour. Moreover, CA survivors appear to report high rates of psychological disorders, with up to 61% experiencing anxiety, 45% experiencing depression, and 27% experiencing post-traumatic stress, including deficits in attention and executive function. These impairments could indicate prefrontal cortex dysfunction and/or biochemical alterations in mesocorticolimbic circuitries (Green et al., 2015). To our knowledge, no studies have assessed the effects of GCI on impulsivity or behavioural disinhibition.

Impulsivity can be divided into subtypes of inhibitory control, which are largely independent (Bari and Robbins, 2013). Of interest, impulsive choice, or the inability to defer gratification, is often observed in pathological conditions and addiction studies (Amlung et al., 2017; Noda et al., 2020; Scherrer et al., 2020). It is frequently studied in humans and animals using delay discounting (DD) paradigms, which offer a decision between a smaller reward delivered immediately (smaller, sooner; SS) and a larger reward presented at progressively longer delays (larger, later; LL; Mar and Robbins, 2007). As the delay for reward increases, its perceived value decreases, illustrating the concept of *delay discounting* (Frost and McNaughton, 2017). Rodents favouring smaller rewards with a short delay over longer-awaited larger rewards display choice impulsivity. While no studies have, to our knowledge, assessed DD following GCI, Déziel and Tasker (2017) reported a stronger preference for SS rewards following endothelin-induced ischemic lesions to the orbitofrontal cortex, compared to non-lesioned or medial prefrontal cortex lesioned rats. Combining classic behavioural tests having hinted at behavioural impulsivity/disinhibition with a DD task could allow for a distinction between components of impulsivity affected by GCI. In this optic, exposure to a naturally aversive stimulus, such as a predator’s urine or faeces (Storsberg et al., 2018) has been associated with raised CORT secretion and anxiety in rodents (Apfelbach et al., 2005; Thomas et al., 2006) and has not been employed in an ischemic sample. This is of interest since ischemic rats have shown decreased freezing in the event of naturally-aversive foot shocks in a fear conditioning paradigm (de la Tremblaye and Plamondon, 2011). Exploration patterns and interactions with this fear-inducing stimulus could allow further characterisation of the ability to appraise anxiogenic stimuli and behavioural disinhibition in ischemic rodents.

The mesocorticolimbic pathway has been shown to be critical in regulating impulse control, largely through dopamine (DA)-ergic activity (Beaulieu and Gainetdinov, 2011). Notably, postsynaptic DA receptor D_2_ (DRD_2_) density within this pathway is associated with elevated impulsivity (Dalley et al., 2007; Besson et al., 2013; Mitchell and Potenza, 2014), while increased DA release mediated by dopamine transporters (DAT) is linked with willingness to exert more effort to receive gratification in reward discounting paradigms (Castrellon et al., 2021). Furthermore, mesolimbic overexpression of ΔFosB, a transcription factor for neuronal activation, is associated with sustained search for gratification following repeated reward-related tasks (Nestler et al., 2001). Of interest, ischemia induces profound alterations of mesocorticolimbic DA activity. Studies have supported behavioural impairments to partly result from a massive post-ischemic DA release (Kawano et al., 1988; Chang et al., 1993; Toner and Stamford, 1996; Kronenberg et al., 2012; Ruscher et al., 2012) after which DA availability, along with DRD_2_ and DAT, are significantly reduced (Takagi et al., 1995; Martín et al., 2013; Momosaki et al., 2017; Uzdensky et al., 2017). Considering impairments in response inhibition in other contexts and the importance of DA signalling in modulating reward-directed behaviour, it is reasonable to think changes to post-ischemic DA-ergic disruptions could be involved in impaired impulse control.

Therefore, the goals of this study were to assess whether reduced anxiety-like behaviours in the EPM and OFT recorded after ischemia could indicate larger impairments in behavioural disinhibition. To do so, impulsive choice in a DD paradigm and defensive behaviours in the Predator Odour Test (PO) were evaluated. This study also assessed changes in the expression of DRD_2_, DAT, and ΔFosB in the basolateral amygdala (BLA), nucleus accumbens (NAc), core (NAcC) and shell (NAcS), and ventromedial prefrontal cortex (vmPFC), which are closely linked to DD and decision-making (Cardinal et al., 2001; Winstanley et al., 2004; Churchwell et al., 2009; Valencia-Torres et al., 2012; Hiser and Koenigs, 2018), to gain a better understanding of underlying mechanisms in the mesocorticolimbic pathway of ischemic rodents. We hypothesised that GCI would impair inhibitory control in both tasks mentioned above, and that these changes would be reflected by lower DRD_2_ and DAT expression, and overexpression of ΔFosB in the mesocorticolimbic pathway.

## Materials and Methods

### Subjects

Eighteen male Long Evans rats were obtained from Charles River Laboratories (Rochefort, Québec, Canada) and arrived at the facility on postnatal day (PND) 21. Upon arrival, rats were housed individually in plexiglass cages containing beta chip bedding and a 3.5-inch diameter black polycarbonate tube, which provided a secure area to rest and hide. Rats were kept on a 12 h light:dark cycle (lights on at 6 a.m.), with all experiments completed during the light photoperiod. Room temperature was maintained at 21–23°C, and relative humidity was held at 60%. Rats had *ad libitum* access to water throughout the experiment.

**FIGURE 1:**
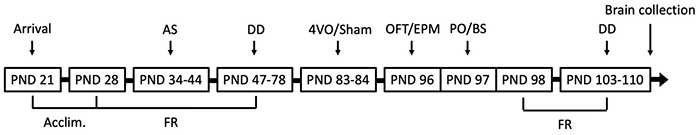
Experimental timeline. Rats were acclimated for 7 days prior to start of food restriction (FR). Autoshaping (AS) and delay discounting (DD) occurred 5 days per week, for 9 and 24 days, respectively. Four-vessel occlusion (4VO) and sham (Sh) surgeries were performed when rats were 83 and 84 days old. Behavioural testing took place following 11 days of recovery and consisted of the Open Field Test (OFT), Elevated-Plus Maze (EPM), and Predator Odour Test (PO) with blood sampling (BS), after which FR was resumed. Rats completed six DD sessions and were perfused immediately after the last session. PND, Postnatal Day.

*Ad libitum* access to standard rat chow (2018 Teklad global 18% Protein Rodent Diet, Envigo, United States) was given during the seven-day acclimation period, after which rats were placed on a food restriction (FR) regimen (14 g of chow/day). Autoshaping training began on the 7*^th^* day of FR. After each DD session, daily food allocation was adjusted by subtracting the weight of sucrose pellets consumed during the session (0.045 g/pellet) from the total food allowance of 14 g. *Ad libitum* access to chow was reinstated 5 days prior to surgery and maintained until 6 days before start of DD sessions (see Figure 1 for experimental timeline). FR protocol was determined by a pilot study performed in our laboratory and aimed at maintaining subjects at 85% of normal body weight, serving as motivation for participation in the DD task. Rats were weighed daily to ensure FR was not interfering with healthy growth. All procedures were performed in accordance with ARRIVE guidelines and the Canadian Council on Animal Care and were approved by the Animal Care Committee of the University of Ottawa.

### Delay Discounting Paradigm

The DD paradigm was modelled after previous research (Cardinal et al., 2001; Winstanley et al., 2006b; Zeeb et al., 2010), with some modifications to length of delays (optimisation validated by the previously mentioned pilot study). All sessions took place between 10 a.m. and 3 p.m., from Monday to Friday. Rats were placed in a quiet, enclosed room for 30 min prior to the start of each session to habituate to the testing area. Task was completed using eight operant conditioning chambers (25.5 cm × 30.5 cm × 32.5 cm; HABITEST Modular Behavioral Test System, Coulbourn Instruments LLC, United States) controlled by the Graphic State 3.03 software (Coulbourn Instruments LLC, United States). Each box consisted of two retractable levers placed on either side of a central food magazine, in which sucrose pellets (5TUT Sucrose Reward Tablets 45 mg – Chocolate, TestDiet, St. Louis, MO, United States) were delivered by a pellet dispenser. The chamber contained two light sources – one within the food magazine (traylight) and one attached to the chamber’s ceiling (houselight). Each chamber was kept in a ventilated, sound-attenuating box to mask any external noises or sources of light. Operant boxes were thoroughly cleaned with 70% ethanol between each use.

#### Autoshaping

Rats learned to associate lever presses with food rewards using a fixed reward ratio of 1. The session consisted of a 40-s “Start” period, followed by two blocks, each comprising 50 trials (30 s/trial). The house light remained illuminated for the duration of autoshaping (AS) sessions. Following the “Start” period during which both levers were retracted, Block 1 began with presentation of the first lever. A lever press within 10 s led to a flash of the tray light and immediate delivery of a single sucrose pellet. After the pellet was delivered, or if rats failed to press the lever within 10 s, rats entered a 30-s intertrial interval (ITI), during which both levers were retracted. After 50 presentations of the first lever, Block 1 was completed, and Block 2 was immediately initiated with the presentation of the second lever. The order of lever presentation was counterbalanced between subjects. The rats completed 9 days of AS training prior to DD task.

#### Delay Discounting

**FIGURE 2:**
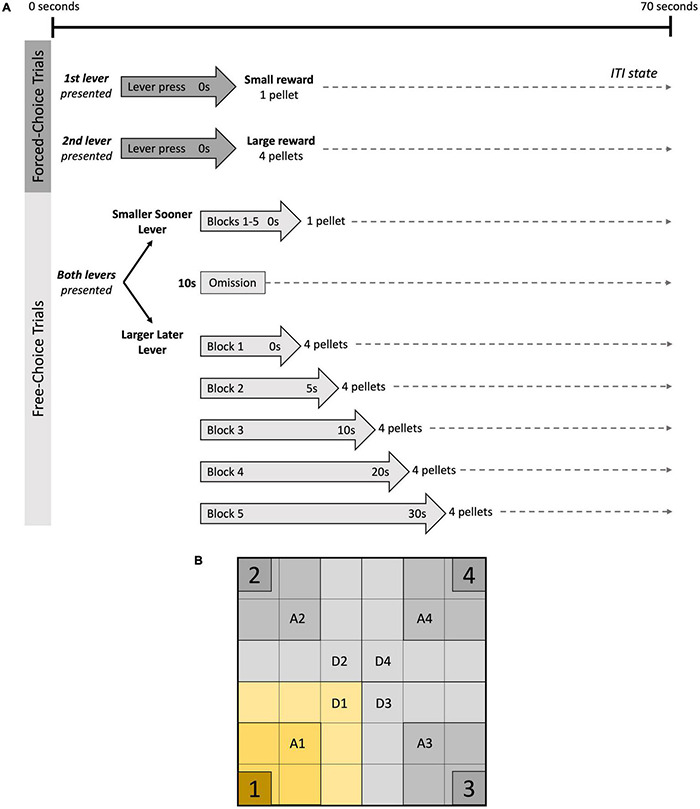
DD task and PO arena. **(A)** Delay discounting (DD) task: each block consisted of two forced-choice trials, followed by 10 free-choice trails. During forced-choice trials, only one lever was presented at a time, and corresponding food reward was dispensed if lever was pressed within 10 s, followed by intertrial interval (ITI), during which both levers were retracted. In free-choice trials, both levers were presented. Lever press led to delivery of corresponding food reward (1 or 4 sucrose pellets), after which the ITI was initiated for the remainder of the trial. Delays for large reward increased after each block (0, 5, 10, 20, and 30 s). Failure to press a lever within 10 s was considered an omission and led to ITI. **(B)** Predator odour (PO) Test arena: for analysis, each quadrant was divided into zones which were adjacent (A1–A4) and distant (D1–D4) to the clear plastic containers placed in each corner. Container #1 held the bobcat urine.

Each session consisted of a 40-s “Start” period with the house light illuminated and both levers retracted, followed by five blocks of 12 trials (70 s/trial). Each block began with two forced-choice trials. During the first forced-choice trial, the house light was illuminated, and the first lever presented. A lever press led to immediate delivery of a small reward (1 sucrose pellet), with the tray light illuminated and house light extinguished. After reward delivery, or if the lever was not pressed within 10 s, rats entered the ITI, during which both levers were retracted, and all lights were extinguished until the next trial. The second forced-choice trial was identical to the first, with the exception of the second lever being presented, leading to immediate delivery of a large reward (4 sucrose pellets) if pressed. Ten free-choice trials followed, during which the house light was illuminated and both levers were presented. Lever presses led to immediate delivery of associated food reward (small or large) and initiation of the ITI stage. Omissions after 10 s led to ITI stage until the end of trial. After 10 free-choice trials, Block 2 was initiated. All 12 trials were repeated identically, except that large reward delivery now occurred after a 5-s delay following lever press (free-choice trials), with tray light illuminated until the four pellets were presented. Each consecutive block followed the same pattern, with the LL reward being increasingly delayed: 10, 20, and 30 s for Blocks 3, 4, and 5, respectively (see Figure 2 for visual representation of the DD task). Small and large reward lever placement was counterbalanced between subjects. The rats completed 24 days of DD training prior to GCI surgery and 6 days of DD post-surgery.

### Transient Forebrain Ischemia

Global cerebial ischemia (GCI; *n* = 9) was induced using the four-vessel occlusion (4VO) model as previously described (Pulsinelli and Brierley, 1979; Pulsinelli and Buchan, 1988). After induction of anaesthesia by inhalation of 4% isoflurane dissolved in 1.5 L/min O_2_ (maintained at 1.5% during surgery), rats were placed in prone position in a stereotaxic apparatus and a 1.5-cm incision was made on the back of the neck. Bilateral alar foramina were exposed and irreversibly electrocauterised (3–4 mV current; MV-8 Veterinary Electrosurgical Unit, Macan Manufacturing, United States). The incision was closed with surgical clips, and the rats were moved to a supine position. A 1.5-cm vertical incision made above the animal’s thorax allowed isolation of the common carotid arteries using loose small-diameter silk thread. Incision was closed and rats were allowed overnight recovery. The following day, ventral incision was reopened under light anaesthesia. Then, bilateral common carotid arteries were reversibly occluded with microvascular clamps for 10 min in spontaneously ventilating rats. Absence of righting reflex and lack of response to light and sound confirmed achievement of ischemic state. Control sham animals (*n* = 9) underwent the same anaesthesia and surgical incisions as GCI rats, without electrocautery of alar foramina or microvascular clamping of common carotid arteries.

Analgesia protocol began on first day of surgery and consisted of two daily (6:30 a.m. and 2:30 p.m.) sublingual buprenorphine (Bup; 0.4 mg/kg) doses for three consecutive days. Bup pellets were crushed and evenly dissolved in hazelnut paste (Nutella©). Appropriate dosage was spread onto a small piece of surgical tape and stuck to the cage wall until ingestion. This analgesia delivery method has been used previously and has the advantage of not interfering with the ischemic process (Kalliokoski et al., 2010; Morin et al., 2021). If the dose had not been consumed by the time the next dose was to be given, a subcutaneous Bup HCl injection (0.05 mg/ml; Vetergesic© Multidose, Ceva Animal Health, Canada) was administered instead. Animals were kept on surgical heating platforms during surgeries (Prostation Rodent Workstation, Patterson Scientific, United States) and were placed in a heated incubator (32°C) immediately after each procedure. Subcutaneous saline injection (5 ml) followed the first procedure and wet rat chow and hydrogel (DietGel© Recovery, Clear H2O, United States) were provided after both.

### Behavioural Tests

Behavioural testing began after 11 days of recovery. The OFT, EPM, and PO were conducted between 7 a.m. and 2 p.m., whereas six post-operative DD sessions were performed at the same times as the pre-operative sessions (between 10 a.m. and 3 p.m. see Figure 1). Prior to testing, rats were placed in a secure, quiet room for 30 min to habituate to the testing area. An overhead analogue camera (WV-CP284, Panasonic, Canada) was used to record behaviours. Seeing as lighting conditions are known to play an important role in modulating behavioural responses (Milot and Plamondon, 2008), the testing area was brightly illuminated (300 lux). All testing apparatus were thoroughly cleaned between each use with 70% ethanol (EPM) or Quato (OFT/PO; 1.6 ml Quato/100 ml water; Swish^®^ Quato™ 44 General Purpose Disinfectant, Swish^®^, Peterborough, ON, Canada).

#### Open Field Test

The OFT was used to measure locomotor activity and exploratory or anxiety-like behaviours (Seibenhener and Wooten, 2015) and served as the habituation phase for the PO (Storsberg et al., 2018). The testing area was surrounded by white walls, and the researcher was hidden behind a white curtain. The apparatus was kept on a table raised 75 cm above ground and consisted of a grey floor surrounded by walls (LWH: 75 cm × 75 cm × 46 cm). The testing area surface was a painted grid of 36 equally sized squares. The peripheral zone was defined as the 20 external squares, whereas the central, more anxiogenic zone represented the 16 innermost squares. Rats were placed in one corner of the OFT, facing the central zone, and left to explore the testing area for 10 min, after which they were returned to their home cage. Noldus© EthoVision XT 7.1 software (Noldus, Leesburg, VA, United States) was used to track distance travelled in central and peripheral zones (cm), ratio of time spent in central and peripheral zones, and latency to enter central zone (s). The number of rearing behaviours in the central and peripheral zones, and time spent grooming in central and peripheral zones (s) were scored by a blinded examiner.

#### Elevated-Plus Maze

One hour after completion of the OFT, the EPM was used as a measure of anxiety-like behaviours (Rico et al., 2016) and behavioural disinhibition. The apparatus consisted of a cross-shaped platform elevated 60 cm above ground. The centre of the platform was a square (LW: 10 cm × 10 cm), to which were attached two open arms (LW: 50 cm × 10 cm) and two closed arms surrounded by walls (LWH: 50 cm × 10 cm × 40 cm). Rats were placed in the centre square facing one of the open arms and left to explore for 5 min, before being returned to the home cage. During testing, subjects were hidden from the researcher by an opaque white curtain. Noldus© EthoVision XT 7.1 software was used to track time spent in the centre zone, open arms and closed arms (s) [(time in arm or zone/(time in open + closed arms + centre zone)) × 100], number of open and closed arms entries, crossings (defined as rats going from one open or closed arm directly into the other open or closed arm, respectively), and latency to enter open arm (s). A blinded examiner scored the number of risk assessment behaviours (each time a rat placed both forepaws into the central zone while remaining in a closed arm) and head dips (each time a rat looked over the edge of an open arm).

#### Predator Odour Test

The PO protocol was performed over two consecutive days, the first being the 10-min OFT serving as a habituation session where baseline exploration data was collected. The following day, the PO session aimed to assess defensive behaviour and appraisal of fear-based stimuli by exposing rodents to a predator odour within the previously habituated OFT arena (Storsberg et al., 2018). Four clear containers (LW: 12.5 cm × 12.5 cm) were placed in each corner of the arena, each containing a small piece of filter paper (LW: 2 cm × 2 cm), one of which had been sprayed with 3 ml of bobcat urine (Maine Outdoor Solutions, United States). Rats were placed in the centre of the arena facing the container with the urine-spayed filter paper and were left to explore for 10 min. All pieces of filter paper were replaced between subjects. For statistical analyses, the arena was divided into 4 quadrants, which were subdivided into two zones: the “Adjacent” zone containing the clear plexiglass containers, and the “Distant” zone, which bordered the Adjacent zone (Quadrant 1 containing the bobcat urine, Quadrants 2 and 3 being adjacent to Quadrant 1, and Quadrant 4 being opposite to Quadrant 1; see Figure 2 for visual representation). Noldus© EthoVision XT 7.1 software was used to determine the distance travelled (cm) and the time spent in each zone (s), along with latency to enter each Adjacent zone (s). Avoidance behaviour was determined by comparing time spent in each zone during the habituation phase (OFT) and during the PO. A blinded examiner scored the number of entries into each Quadrant, percent time in direct interaction with each plexiglass container (defined as s of physical contact with containers), and direction of approach into the Adjacent zones [defined as approaching from the squares near the outer walls (peripheral approaches) or from the centre of the arena (central approaches)]. Additionally, freezing behaviours (defined as 3 s without movement), time spent grooming (s), and rearing behaviours were measured to quantify more discreet displays of anxiety-like/defensive behaviours.

#### Blood Sampling

Blood samples were obtained on the day of PO testing to provide a measure of circulatory CORT concentrations before and after exposure to an anxiogenic stimulus. Collection was done by nicking the tail vein (Milot et al., 2012) and depositing two drops of blood onto Whatman Bloodstain Cards (Whatman International Ltd., Maidstone, United Kingdom). Blood samples were taken 15 min before (baseline) as well as 0, 30, and 120 min after the PO. Specimen collection paper was left to dry overnight, then stored at −80°C until assay.

### Blood Corticosterone Immunoassay

Using the samples collected during the PO, blood drop CORT concentrations were analysed via enzyme-linked immunosorbent assay (ELISA; Corticosterone ELISA Kit, ADI-900-097, Enzo Life Sciences, Inc., Farmingdale, United States) as validated by Milot et al. (2012). Whatman cards were removed from the −80°C freezer and were left to thaw at room temperature for 30 min before a Gem Hole Punch (McGill Inc., Marengo, IL, United States) was used to punch samples into glass tubes. Assay buffer (200 μl) was pipetted into every tube, which were then sealed with parafilm and left for 24 h on a Belly Dancer^®^ (Structure Probe Inc., West Chester, PA, United States). The following day, 2.5 μl of steroid displacement reagent was added to 100 μl of sample solution and were left to rest for 5 min at room temperature. Then, 200 μl of assay buffer was added to samples which were then centrifuged at 5,000 *g* for 30 s. Afterwards, 100 μl of each sample were pipetted in appropriate plate wells followed by 200 μl of alkaline phosphatase conjugated with CORT and sheep polyclonal antibody to CORT. The plate was covered and left on a plate shaker for 2 h at 500 rpm. The wells were then rinsed three times with wash buffer, 200 μl of pNpp solution was added, and the plate was covered and left to incubate for 45 min at room temperature. A stop solution was added then the plate was placed on a plate shaker at 500 rpm for 30 s before being scanned using a PowerWave XS2 Microplate Spectrophotometer (BioTek, United States). Assay detection range was 32–20,000 pg/ml and intra and inter-assay consistency was confirmed by measuring the coefficient of variability and normalising detection levels for all plates, respectively.

### Brain Tissue Collection

Immediately following the last DD session, rats were injected with a lethal dose of sodium pentobarbital (60 mg/ml; Euthanyl Bimeda-MTC Canada, Canada) and were transcardially perfused (4% paraformaldehyde; 20% picric acid). Brains were extracted and incubated in paraformaldehyde for 1 h, 10% sucrose for 1 h, then incubated in 10% sucrose overnight. The next morning, brains were incubated in 10% sucrose for 1 h, after which they were frozen with CO_2_ and stored at −80°C. Subsequently, coronal brain slices (14 μm) were obtained using a cryostat (Leica CM1900, Leica Microsystems, Germany) and mounted onto Superfrost Plus Slides (Fisher Scientific, Canada). In this study, regions of interest were the BLA (Bregma −1.60 to −2.12 mm), hippocampus CA1, and CA3 (Bregma −2.80 to −4.16 mm), NAcC and NAcS (Bregma 1.60 to 0.70 mm), and vmPFC (Bregma 2.70 to 1.70 mm). Brain regions were determined using Paxinos and Watson rat brain atlas (Paxinos and Watson, 2006).

### Assessment of Neuronal Cell Death

Thionine staining was used to quantify pyramidal neuron density in the hippocampus CA1 and CA3. Slides were transported in containers filled with 0.01 M phosphate-buffered saline (PBS; pH = 7.4), then soaked in dH_2_O (30 s), thionine (10 min), 50, 70, 95, and 2 × 100% ethanol (2 min each), 2 × Citrisolv (2 min each; Decon Labs, Inc., United States). Slides were covered with Permount Mounting Medium, were cover-slipped, and were left to seal overnight. For each subject, six histologically representative pictures of the CA1 and CA3 hippocampal subregions were obtained using a Leica DAS microscope with an attached SONY digital camera and were recorded *via* Norton Eclipse 6.0 software (Empix Imaging, Mississauga, ON, Canada). Pyramidal neuron density within 1 mm linear length was quantified with ImageJ software (National Institutes of Health, United States) by two blinded examiners with achievement of inter-rater reliability. Intact neurons were defined as having a clear nuclear area enclosing a nucleolus and a cytoplasm contained within a rounded cell body.

### Fluorescence Immunohistochemistry

For ΔFosB and DAT fluorescence immunochemistry, slides containing the regions of interest were transported in 0.01 M phosphate-buffered saline (PBS) and incubated for 30 min in blocking solution (5% donkey serum–0.2% triton–PBS). The slides were incubated overnight at 4°C in blocking solution containing monoclonal mouse anti-ΔFosB (1:2000; ab11959, Abcam, Canada – this antibody stained for both FosB and ΔFos) and monoclonal rabbit anti-DAT (1:1000; ab128848, Abcam, Canada) primary antibodies. After incubation, slides were washed in PBS (3 washes × 5 min) and incubated in a blocking solution containing fluorescence-conjugated anti-mouse donkey (1:1000; A-21202, Thermo Fisher Scientific, Waltham, MA, United States) and anti-rabbit donkey (1:1000; A-21207, Invitrogen Canada Inc., Canada) secondary antibody for 1 h at room temperature (RT) in a dark cabinet. Slides were washed in PBS (3 washes × 5 min), incubated in Hoechst adenine-thymine binding dye solution (1:20,000 in PBS; Hoechst 33342, Invitrogen Canada Inc., Canada) for 5 min at RT, and PBS washed (3 washes × 5 min). Slides were coated with 30 μl anti-fading solution, cover slipped, and sealed with nail polish. Negative controls (without primary antibody) ensured secondary antibody specificity and pilot studies determined optimal primary antibody concentrations. Staining and expression was similar to previous studies (See Larson and Ariano, 1995 for DRD_2_; Sun et al., 2020 for ΔFosB; and Threlfell et al., 2021 for DAT). For DRD_2_, antigen retrieval was performed prior to blocking. Briefly, slides were incubated in 0.05 M sodium citrate-PBS (30 min), rinsed in PBS (3 washes × 5 min), soaked in 0.1 M Glycine-PBS (30 min), then PBS washed again (3 washes × 5 min) before the continuation of the previously described protocol. Of note, incubation in monoclonal rabbit anti-DRD_2_ primary antibody (1:500, ab1558, Millipore, United States) lasted 24 h at room temperature, followed by three 10-min PBS washes. The secondary antibody was the same as that for DAT (1:1000).

Fluorescence was detected using an Olympus DX51 microscope (Center Valley, PA, United States) using a 20× magnifier. Six anatomically matched pictures were taken for each structure using Progress Pro 2.7.6 software (Jenoptik, Jena, Germany). ImageJ software (National Institutes of Health, United States) was used to quantify percentage of immunoreactivity relative to total photomicrograph area (percentage of area) and ratio between the mean immunoreactive foreground and subthreshold background (grey value; Jensen, 2013). Higher percentage of area represents larger area covered by staining, whereas mean grey value measures signal intensity related to staining density. Auto-threshold algorithm Triangle was used for all structures and markers, except for DAT expression in the NAc and BLA, for which Moments was used.

### Statistical Analyses

All analyses were completed using IBM© SPSS Statistics 27 (IBM, Armonk, NY, United States). Outliers were identified by box plots and were corrected by adding or subtracting a value of one to the second most extreme data point within that group (or a value of 0.01 when data points were ratios). Homogeneity of variance and normality were assessed with Levene’s and Shapiro-Wilk’s tests, respectively. Data for DD were combined in 3-day clusters, with last three preoperative and last three postoperative sessions being used for statistical analyses. Area under the curve (AUC) was calculated for each subject using the following equation (*x*_2_−*x*_1_)[(*y*_1_+*y*_2_)/2] (Myerson et al., 2001). AUC data was used in a two-way mixed analysis of variance (ANOVA) with surgical status (preoperative vs. postoperative) as within-subject factor and group (sham vs. GCI) as between-subject factor. LL lever choice ratio was calculated by dividing the number of LL lever presses by total lever presses for that block, which was then multiplied by 100. Missing data points (*n* = 2; Block 5, one sham, one GCI) were computed by multiple imputation. Effect of delay on LL lever preference was assessed with a three-way mixed ANOVA with blocks (Blocks 1–4) and surgical status (preoperative vs. postoperative) as within-subject factors and group (sham vs. GCI) as between-subject factors. Nonparametric Friedman’s test was used to analyse omissions in the DD task. Behavioural (OFT, EPM, PO), thionine, and fluorescence immunohistochemistry data were analysed using independent samples *t*-tests or Welch’s *t*-test if homogeneity was violated. PO avoidance data was analysed using a two-way mixed ANOVA, with session (habituation vs. PO) as within subject-factor and surgery (sham vs. GCI) as between-subject factor while ELISA data was analysed using a two-way mixed ANOVA, with time (baseline, 0, 30, 120 min post-test) as within-subject factor and surgery (sham vs. GCI) as between-subject factor. Multiple imputation was used for a few missing ELISA data points. Pairwise comparisons were analysed with Bonferroni correction. Statistical significance was set at *p* < 0.05.

## Results

All rats included in the GCI group (*n* = 9) achieved ischemic state, as confirmed during surgery and through neuronal density assessment. One sham subject could not be included in thionine and fluorescence immunohistochemistry analyses due to technical issues (sham: *n* = 8).

### Delay Discounting

**FIGURE 3:**
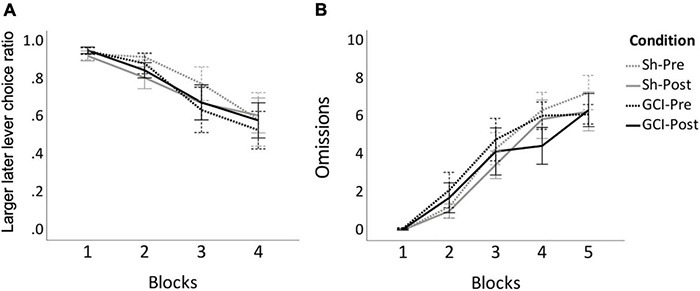
DD before and after GCI. Figure shows results of sham (Sh) and global cerebral ischemia (GCI) rats pre- and post-surgery for larger later (LL) lever choice ratio **(A)** and number of omissions **(B)** during the 10 free-choice trials of each block of the delay discounting task. Choice ratio for Block 5 was excluded due to large number of omissions. Choice ratio for LL lever significantly decreased over blocks (*p* < 0.0005), whereas omissions significantly increased over blocks (*p* < 0.001). Values are presented as the means of last three preoperative sessions (Pre) and last three postoperative sessions (Post) for each group, ±S.E.M.

Two-way mixed ANOVA of AUC data revealed no significant difference in overall LL lever preference between groups or surgical status (*p* > 0.05; see Figure 3). Three-way mixed ANOVA revealed a significant main effect of blocks on choice ratio [*F*(3,48) = 20.338, *p* < 0.0005, η^2^_p_ = 0.560]. Pairwise comparisons found choice ratio during Block 1 to be significantly higher than that of Blocks 2 (*p* = 0.002), 3 (*p* = 0.001), and 4 (*p* < 0.0005). During Block 2, the preference for LL lever was significantly higher than during Blocks 3 (*p* = 0.011) and 4 (*p* = 0.005). All rats preferred the LL reward when the delay was short but showed increased preference for the SS reward as LL delays increased. Due to low participation rate, the 5*^th^* block was excluded from choice ratio analysis, being nonrepresentative of the overall performance.

As no between-group differences were reported for the area under the curve and choice ratio, omissions data from sham and GCI rats were analysed together. Friedman’s test revealed that omissions differed significantly between blocks [χ^2^(9) = 112.176, *p* < 0.001] (see Figure 3). Prior to surgery, pairwise comparisons showed omissions during Block 1 to be significantly lower than during preoperative Blocks 3 (*p* = 0.001), 4 (*p* < 0.0005), and 5 (*p* < 0.0005), while omissions during Block 2 were significantly lower than during Block 4 (*p* < 0.0005) and 5 (*p* < 0.0005). Similarly, post-operatively, *post hoc* analyses showed significantly less omissions during Block 1 than Blocks 3 (*p* = 0.004), 4 (*p* < 0.0005), and 5 (*p* < 0.0005). Omissions during Block 2 post-surgery were also lower than postoperative Blocks 4 (*p* = 0.004) and 5 (*p* < 0.0005). No differences between equivalent pre and postoperative blocks were noted (*p* > 0.05).

### Behavioural Tests

#### Open Field Test

**FIGURE 4:**
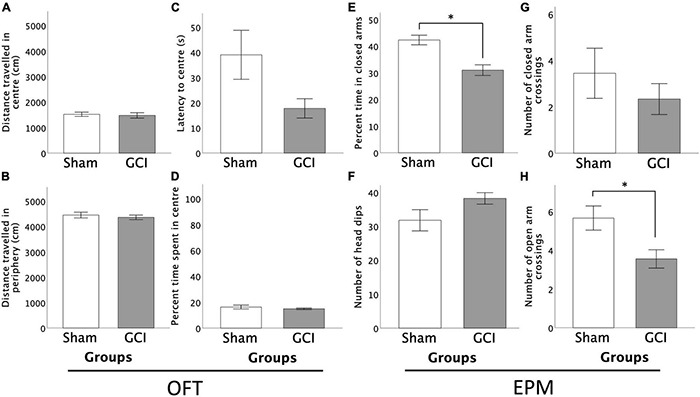
Effects of GCI on OFT and EPM performance. Left side of figure shows distance travelled in the centre **(A)** and periphery **(B)** of the OFT, and latency to centre **(C)** and percentage of time spent in the centre zone **(D)**. Right side of figure shows percentage of time spent in the closed arms of the EPM **(E)**, number of head dips **(F)**, and number of closed **(G)** and open **(H)** arm crossings. Values are presented as mean ± S.E.M. *Indicates statistical significance at *p* < 0.05. GCI, global cerebral ischemia.

As shown in Figure 4, no differences between sham-operated and GCI subjects were found in regard to distance travelled or percentage of time spent in central and peripheral zones of the OFT (*p* > 0.05). However, Welch *t*-test revealed a trend for shorter latency to enter the anxiogenic centrezone in GCI vs. sham-operated rats [*t*(10.427) = 2.035, *p* = 0.068, *d* = 0.959]. There were no differences in grooming or rearing in the central and peripheral zones, and in the number of central or peripheral zone crossings (all *p* > 0.05).

#### Elevated-Plus Maze

As shown in Figure 4, GCI rats spent a significantly smaller percentage of time in the closed arms of the EPM than sham-operated controls [*t*(16) = 4.207, *p* = 0.001, *d* = 1.983]. No differences were found in percent time spent in open arms or centre square, or in number of open or closed arm entries (*p* > 0.05). Sham-operated subjects performed more open arm crossings than GCI rats [*t*(16) = 2.694, *p* = 0.016, *d* = 1.270], but did not differ in number of closed arm crossings (*p* > 0.05). Ischemic rats tended to perform more head dips than sham-operated counterparts [*t*(16) = −1.812, *p* = 0.089, *d* = −0.854]. There were no differences in the number of risk assessments or latency to first open arm entry (both *p* > 0.05).

#### Predator Odour Test

**FIGURE 5:**
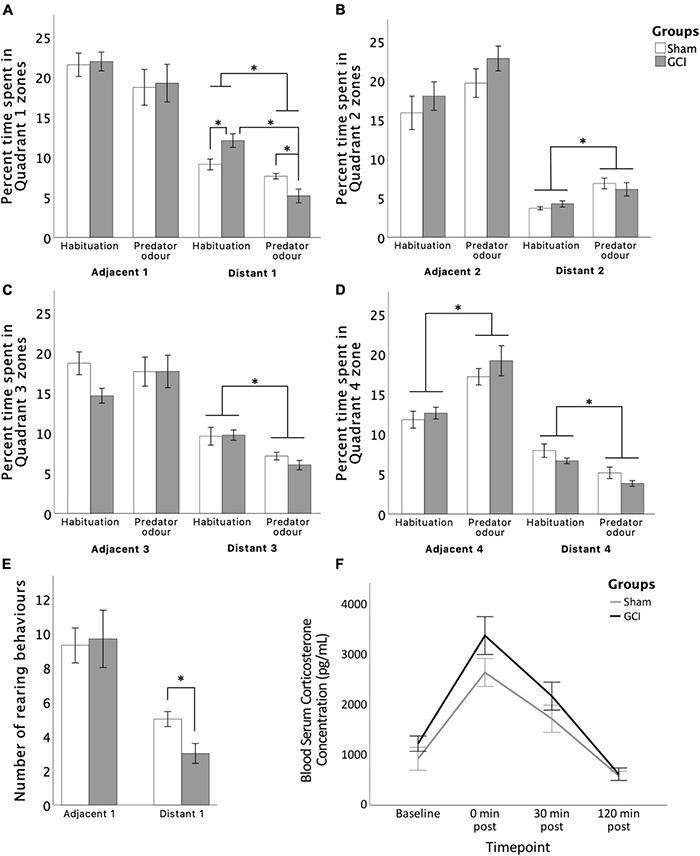
Effects of GCI on PO test performance and circulatory blood corticosterone levels. Bar graphs show time spent in each zone **(A–D)** and number of rearing behaviours in Quadrant 1 **(E)**. Line graph presents blood serum corticosterone levels at baseline and at 0, 30, and 120 min post PO completion **(F)**. Values are presented as mean ± S.E.M. *Indicates statistical significance at *p* < 0.05. GCI, global cerebral ischemia.

Filter paper containing predator bobcat urine was placed in Adjacent 1 zone (Quadrant 1) of the arena (see Figure 2B). Avoidance behaviour was assessed using the time spent in zones during the PO and the habituation phase. Two-way mixed ANOVA showed that during the PO, rats tended to spend less time in Adjacent 1 [*F*(1,11) = 3.998, *p* = 0.071, η^2^_p_ = 0.267] and spent significantly less time in Distant 1 [*F*(1,11) = 37.840, *p* < 0.001, η^2^_p_ = 0.775] than they had during the OFT habituation (see Figure 5). Simple main effects showed that GCI rats spent less time in Distant 1 during the PO than the OFT (*p* =< 0.001), and compared to sham subjects during the PO (*p* = 0.016). However, GCI rats spent more time in Distant 1 compared to sham rats during the OFT (*p* = 0.018). Notably, rats spent an increased amount of time in Distant 2 [*F*(1,11) = 21.858, *p* < 0.001, η^2^_p_ = 0.665] and in Adjacent 4 [*F*(1,11) = 52.502, *p* < 0.001, η^2^_p_ = 0.827], and tended to spend more time in Adjacent 2 [*F*(1,11) = 4.735, *p* < 0.052, η^2^_p_ = 0.301] during the PO than the OFT. Conversely, rats spent less time in Distant 3 [*F*(1,11) = 24.956, *p* < 0.001, η^2^_p_ = 0.686] and Distant 4 [*F*(1,11) = 35.892, *p* < 0.001, η^2^_p_ = 0.765] during the PO than the OFT.

For number of rearings, independent samples *t*-test revealed a significant effect of surgery [*t*(11) = 2.810, *p* = 0.017, *d* = 1.563] with ischemic rats showing reduced rearings in the Distant 1 zone. No significant differences between groups were found for distance travelled, number of entries, and approach patterns in the arena zones (all *p* > 0.05). Inconsistent grooming and freezing scores did not enable valid statistical analysis of those measures.

### Blood Corticosterone Immunoassay

For circulatory CORT levels, two-way mixed ANOVA revealed a significant main effect of time [*F*(1.943,31.087) = 39.378, *p* < 0.0005, η^2^_p_ = 0.711] and surgery [*F*(1,16) = 4.538, *p* = 0.049, η^2^_p_ = 0.221] (see Figure 5). Pairwise comparisons revealed serum CORT levels to be increased immediately following PO (*p* < 0.0005) and to remain elevated 30 min later (*p* = 0.006) when compared to baseline. After 120 min, CORT concentrations had recovered to below baseline levels (*p* = 0.001), possibly due to novelty effect of sampling procedure. Concentrations immediately after and 30 min after completion were both higher than at the 120-min mark (both *p* < 0.0005). Finally, CORT levels recovered gradually, with concentrations immediately after PO being higher than concentrations 30 min post-completion (*p* = 0.02). Overall, serum CORT concentrations were higher in GCI subjects than sham subjects (*p* = 0.049), without a time × surgery interaction (*p* > 0.05).

### Hippocampal Neuronal Injury Assessment

**FIGURE 6:**
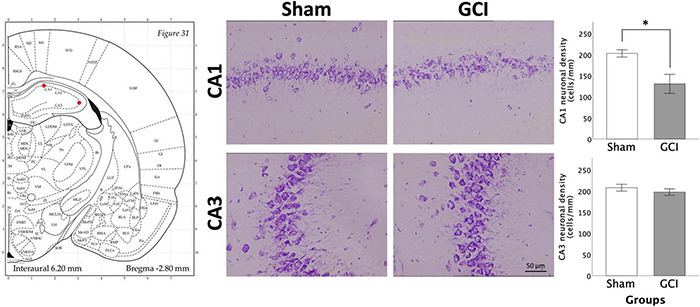
Neuronal injury assessement in the hippocampus CA1 and CA3 regions using thionine staining. Figure shows representative photomicrographs of the CA1 and CA3 subregions of the hippocampus at 20× magnification (1 mm linear length). Histograms present neuronal density in the CA1 and CA3 following sham surgery or GCI, assessed in 14 μm thionine-stained coronal brain slices. Values are presented as mean ± S.E.M. *Indicates statistical significance at *p* < 0.05. Visual representation of rat brain adapted from labs.gaidi.ca/rat-brain-atlas/ (Paxinos and Watson, 2006). GCI, global cerebral ischemia.

Figure 6 shows pyramidal cell density in the CA1 and CA3 for sham and GCI rats. Welch independent samples *t*-test found significant differences in mean neuronal density in the CA1 between sham and GCI subjects [*t*(10.260) = 2.989, *p* = 0.013, *d* = 1.384]. Neuronal density was significantly lower in the CA1 of rats having undergone GCI than in their sham-operated counterparts. There were no significant differences in mean neuronal density in the CA3 (*p* > 0.05).

### Fluorescence Immunohistochemistry

#### Dopamine Transporter Expression

**FIGURE 7:**
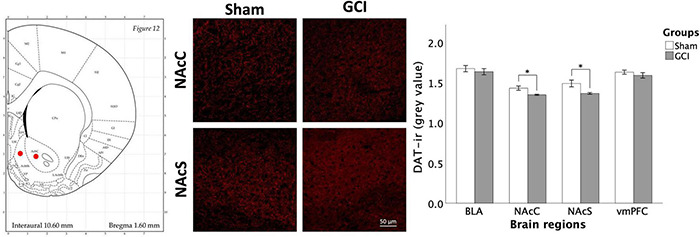
Dopamine transporter fluorescence immunohistochemistry following GCI. Figure shows representative fluorescence photomicrographs of dopamine transporter (DAT) expression at 20× magnification in the nucleus accumbens core (NAcC) and shell (NAcS) of rats having undergone sham surgery or GCI. DAT immunoreactivity is primarily located in dendrites and axons, yielding a net-like expression surrounding the cell bodies. Histogram presents mean grey value for DAT immunoreactivity in each studied brain region. Values are presented as mean ± S.E.M. *Indicates statistical significance at *p* < 0.05. Visual representation of rat brain adapted from labs.gaidi.ca/rat-brain-atlas/ (Paxinos and Watson, 2006). BLA, basolateral amygdala; GCI, global cerebral ischemia; vmPFC, ventromedial prefrontal cortex.

As shown in Figure 7, Welch independent samples *t*-test revealed significant differences for mean grey value in the NAcC [*t*(8.016) = 3.050, *p* = 0.016, *d* = 1.563] and NAcS [*t*(7.982) = 2.843, *p* = 0.022, *d* = 1.458]. Higher DAT mean grey values were observed in sham-operated vs. ischemic rats in both subfields. In contrast, no significant differences in mean grey values were found in the BLA and vmPFC (*p* > 0.05). No significant differences in the percentage of area were detected.

#### Dopamine Receptor D_2_ Expression

**FIGURE 8:**
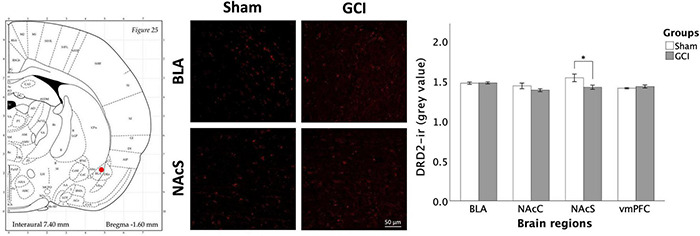
Dopamine receptor D_2_ fluorescence immunohistochemistry following global cerebral ischemia. Figure shows representative fluorescence photomicrographs of dopamine receptor D_2_ (DRD_2_) expression at 20× magnification in the basolateral amygdala (BLA) and NAcS of rats having undergone sham surgery or GCI. Histogram presents mean grey value for DRD_2_ immunoreactivity in each brain region of interest. Values are presented as mean ± S.E.M. *Indicates statistical significance at *p* < 0.05. Visual representation of rat brain adapted from labs.gaidi.ca/rat-brain-atlas/ (Paxinos and Watson, 2006). GCI, global cerebral ischemia; NAcC, nucleus accumbens core; vmPFC, ventromedial prefrontal cortex.

Figure 8 presents DRD_2_ immunoreactivity in the BLA, NAcC, NAcS, and vmPFC of sham and GCI rats. Independent samples *t*-test revealed significant differences in mean grey value in the NAcS [*t*(15) = 2.242, *p* = 0.04, *d* = 1.090]. Sham-operated subjects had significantly higher DRD_2_ density than GCI subjects in the NAcS. There were no differences in percentage of area in the NAcS (*p* > 0.05). Additionally, there were no significant differences in both measures in the BLA, NAcC, or vmPFC (*p* > 0.05).

#### ΔFosB Expression

**FIGURE 9:**
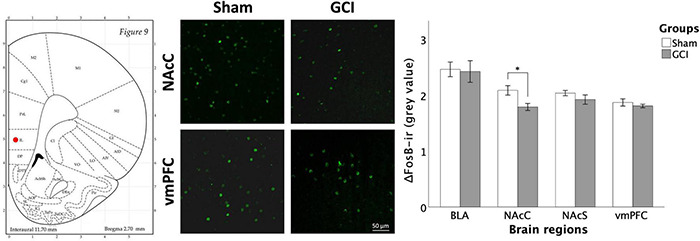
ΔFosB fluorescence immunohistochemistry following GCI. Figure shows representative fluorescence microphotographs of ΔFosB and FosB expression at 20× magnification in the NAcC and ventromedial prefrontal cortex (vmPFC) of rats having undergone sham surgery or GCI. Histogram presents mean grey value for ΔFosB and FosB immunoreactivity in each studied brain region. Values are presented as mean ± S.E.M. *Indicates statistical significance at *p* < 0.05. Visual representation of rat brain adapted from labs.gaidi.ca/rat-brain-atlas/ (Paxinos and Watson, 2006). BLA, basolateral amygdala; GCI, global cerebral ischemia; NAcS, nucleus accumbens shell.

Figure 9 displays ΔFosB immunoreactivity in the BLA, NAcC, NAcS, and vmPFC of GCI and sham-operated subjects. Independent samples *t*-test showed a significant difference in mean grey value in the NAcC [*t*(15) = 2.854, *p* = 0.012, *d* = 1.387], attributable to significantly higher ΔFosB mean grey value in sham compared to GCI rats. Percentage of area did not differ between groups in the NAcC (*p* > 0.05). Independent samples *t*-test also found a trend in percentage of area in the vmPFC [*t*(15) = 1.886, *p* = 0.079, *d* = 0.916] with sham-operated rats trending towards elevated ΔFosB percentage of area compared to GCI rats. Mean grey value did not differ between groups in the vmPFC (*p* > 0.05). There were no significant differences in both measures in the BLA or NAcS (*p* > 0.05).

## Discussion

To our knowledge, this study is the first to: (1) characterise the effects of GCI on impulsive choice by analysing reward selection in a delay discounting paradigm and underlying changes to the dopaminergic mesocorticolimbic system activity regulating such processes and (2) assess alterations to approach-avoidance behaviours in predator odour-induced fear context post ischemia. Collectively, our results do not support GCI to impact impulsive choice in a DD task or defensive behaviour when facing a naturalistic/environmental threat, despite reduced DRD_2_, DAT, and ΔFosB-ir expression within the mesocorticolimbic pathway and significant hippocampal CA1 injury. As previously noted, ischemic rats showed reduced anxiety-like behaviours in the EPM and to a lesser extent, the OFT, which could be indicative of behavioural disinhibition. Decreased propensity of ischemic rats to explore safe zones in the EPM or OFT is not accompanied by increased choice impulsivity and is not linked to impaired ability to appraise fearful or anxiogenic stimuli, supporting differential impacts of GCI on subtypes of response inhibition.

### Global Cerebral Ischemia Does Not Alter Impulsive Choice in a Delay Discounting Task

Using a classic DD paradigm, we showed ischemic and sham rats to both opt for larger rewards when delay to gratification was short, with a gradual shift towards smaller rewards as delay increased. GCI did not accelerate the shift towards a preference for SS reward, contrasting increased preference for the SS reward observed following focal ischemic lesions to the orbitofrontal cortex (Déziel and Tasker, 2017). Our results support damage to vulnerable brain areas such as the hippocampus (Traystman, 2003; Auer, 2016) following 10-min GCI to have a negligible impact on choice impulsivity, possibly salvaging orbitofrontal cortex circuitries. In Wistar rats, reduced brain-derived neurotrophic factor (BDNF), tropomyosin receptor kinase B (TrkB) protein and mRNA expression have been reported in the medial PFC at a longer 30-day interval following GCI (de la Tremblaye et al., 2016), while significant mitochondrial damage and apoptosis in prefrontal cortical tissue were observed 96 h following 20-min global ischemia (Jangholi et al., 2020). Thus, post ischemic delays and intensity of the insults appear to play a critical role in prefrontal cortical dysfunctions.

The well-documented extensive damage to the vulnerable CA1 pyramidal neurons of the hippocampus following GCI (Pulsinelli and Brierley, 1979; Pulsinelli and Buchan, 1988; Bueters et al., 2008) was observed in our study. Interestingly, excitotoxic lesions to the hippocampus have been found to cause impulsive choice in a DD task (Cheung and Cardinal, 2005). However, induced lesions resulted in the destruction of the entire hippocampal structure. Albeit being heavily altered, the CA1 maintains some functional capabilities following GCI and CA3 pyramidal neurons show more resistance to ischemic insult (Knowles et al., 2016). In such context, less direct global ischemia damage to hippocampal structures appears insufficient to affect DD performance. This study being the first to assess DD performance in GCI rats, further research appears necessary to better characterise alterations of decision-making processes post-ischemia and associated neuronal determinants.

### Reduced Anxiety-Like Behaviour in Ischemic Rats Is Not Accompanied by Changes in Defensive Behaviour in the Predator Odour Test

In this study, ischemic rodents displayed reduced time spent in the EPM’s anxiolytic closed arms and a tendency to perform increased head dips into the open arms. They also showed reduced latency to enter the anxiogenic centre zone of the OFT, supporting decreased anxiety-like behaviours. These observations are consistent with studies showing increased exploration of the anxiogenic OFT and EPM zones by ischemic rats, along with decreased latency to explore these zones (Plamondon and Khan, 2005; Yan et al., 2007; Lu et al., 2016). As mentioned, while such a disregard for the anxiogenic properties of the EPM has historically been interpreted as reduced anxiety-like behaviours, a possible link to impaired behavioural inhibition has also been discussed by these authors (Plamondon and Khan, 2005; Milot and Plamondon, 2009). Due to growing literature replicating such results, it has been hypothesised in recent years that this could be indicative of decreased inhibitory control, possibly reflecting behavioural impulsivity (Knowles et al., 2016; Morin et al., 2021). Previous studies have interpreted increased open-arm time in the EPM and decreased latency to the centre of the OFT as potential indicators of impulsivity (Ueno et al., 2002; Binder et al., 2004), although this interpretation requires further investigation (Rico et al., 2019). In the present study, disinhibition was less pronounced than in previous assessments, possibly due to extensive handling of these animals over days related to the DD task. Interestingly however, our demonstration of possible behavioural impulsivity in the absence of changes in DD performance is consistent with Broos et al.’s (2012) comprehensive assessment, which showed impulsive choice and impulsive action to be uncorrelated in both humans and rats. It is important to note that all behavioural testing was conducted under bright light conditions, in which ischemic rats have previously shown increased open-field exploration when compared with sham-operated controls while showing decreased exploration compared to controls when the test was performed under dim light condition (Milot and Plamondon, 2008).

Previous studies have reported exposure to a predator’s urine to act as a psychogenic stressor inducing fear-like and defensive behaviours (Staples, 2010). In this study, we used bobcat urine, shown to elicit the expression of avoidance and defensive behaviours in rats associated with activation of the amygdalo-piriform transition area and subsequent rise in circulatory CORT secretion (Albrechet-Souza and Gilpin, 2019). Our results of decreased time spent in zones nearest to the urine (Adjacent 1 and Distant 1) with increases in zones further away from the target anxiogenic stimulus (Adjacent 2 and 4 and Distant 2) during the PO when compared with the OFT habituation session validate the odour to have induced avoidance behaviours in rats. Supporting the PO to have prompted a stress response, all rats showed sustained hypothalamo-pituitary-adrenal axis activation to the PO stressor, as demonstrated by heightened immediate and delayed CORT expression, with ischemic rats showing higher overall pre- and post-test CORT secretion levels.

Interestingly, GCI rats spent more time in Distant 1 during habituation than sham-operated controls, an outcome reversed by the predatory odour exposure. Furthermore, ischemic insult decreased the number of rearings performed in the Distant 1 zone during the PO session. These results demonstrate, to an extent, a propensity for ischemia to induce an anxiogenic, fear-based response when confronted with survival-associated stimuli. Our findings also showed comparable responses on other PO measures (number of entries, latency to odour, time in direct interactions, and approach patterns) in sham and ischemic rats. This is consistent with locomotor responses to weasel odour being unaffected following colchicine-induced hippocampal lesions in rats (Perrot-Sinal and Petersen, 1997). The differences in approach responses by ischemic animals in presence or absence of a predator odour support cognitive appraisal of fear-specific cues, as opposed to anxiety-related ones, to be preserved after ischemia. Thus, such results further indicate the behavioural disinhibition witnessed in the EPM/OFT to be context specific. Future studies assessing differences related to odour specimen and/or rodent strains are required to further understand the impact of GCI on adaptive coping strategies when facing environmental threats (Adduci et al., 2021).

### Global Cerebral Ischemia and the Mesocorticolimbic Pathway

Dopamine (DA)-ergic pathway function is known to be greatly impaired in the weeks following brain infarction, as shown by decreased DA availability (Takagi et al., 1995), and both DRD_2_ (Gower and Tiberi, 2018) and DAT (Yanagisawa et al., 2006) expression. These observations are consistent with reduced DRD_2_ and DAT immunoreactivity in this study. Levels of tyrosine hydroxylase, the rate-limiting enzyme in DA synthesis, have also been shown to fluctuate post-ischemia, being decreased 9 days after GCI (Knowles et al., 2016) while being upregulated at the 30-day mark (de la Tremblaye et al., 2014). This is also concordant with our observed DRD_2_ downregulation.

With alterations to DA-ergic function confined to the NAc and with reduced NAcC activation, as shown by decreased ΔFosB expression, our results support the vulnerability of this structure to ischemia. Considering a crucial role of the NAc in regulating impulsive choice (Cardinal et al., 2004; da Costa Araújo et al., 2009), the absence of changes to DD performance in GCI rodents is surprising. Interestingly, uncovered distinctions in the roles of the NAcC and NAcS suggest that the location of impaired DA-ergic function within this structure may contribute to our observations. Whereas lesions to the NAcC usually increase DD (Cardinal et al., 2001), lesions to the NAcS produced no effects on the same task (Pothuizen et al., 2005). Furthermore, injections of the DRD_2_ antagonist eticlopride into the NAcC decreased self-administration of both cocaine and food, whereas injection into the NAcS did not influence food self-administration (Bari and Pierce, 2005). As such, lowered DRD_2_-ir in the NAcS alone may not be sufficient to induce impulsive choice.

The dual action of DAT in removing DA from the synapse and repackaging it for future release complexifies identifying a defined role in regulating impulsive choice. In a meta-analysis by Castrellon et al. (2021), modulation of DAT activity was shown to lessen discounting behaviours, likely as a result of heightened DAT-mediated DA release. In contrast, Adriani et al. (2009) found that accumbal injections of a DAT gene enhancer or DAT silencer had similar impact in a DD task, with both drugs reducing the preference for the LL reward, with a slightly more pronounced effect from DAT over expression. The authors concluded that modifications to DAT function, whether through enhancing or silencing, altered inhibitory self-control. As such, the fact that reduced NAc DAT density in GCI rats was not associated with heightened impulsive choice is surprising. Although mechanisms remain to be determined, strong connections exist between the NAc and BLA (Winstanley et al., 2006a) and DAergic function in the BLA has not been altered by GCI in this study, which could have contributed to counteract effects of post-ischemic NAc dysregulations.

Furthermore, research has supported ΔFosB expression to be associated with sensitisation to the motivational properties of drugs of abuse, thus acting as a “molecular switch” involved in maintaining addiction (Nestler et al., 2001). In a study on food addiction-like behaviours, high impulsivity rats had increased ΔFosB expression in the NAcS compared to low impulsivity groups, irrespective of having access to regular rat chow or a highly palatable diet. In contrast, ΔFosB expression in the NAcC was increased in both highly palatable diet groups but did not vary between impulsivity groups (Velázquez-Sánchez et al., 2014). These findings further support differential roles of the NAc subregions in modulating impulsive behaviours and are consistent with an absence of changes in post-GCI impulsive choice in the presence of reduced ΔFosB expression at the NAcC. To our knowledge, our study is the first to measure ΔFosB expression at a remote 26 days after GCI. Our observations of reduced ΔFosB immunoreactivity contrast with shorter term assessments which found ΔFosB to be overexpressed 48 h after ischemia (McGahan et al., 1998; Kurushima et al., 2005) and to return to near basal levels after 1 week (Kurushima et al., 2005).

Interestingly, while discrete changes to DA-ergic function in the mesocorticolimbic pathway following GCI are not sufficient to increase impulsive choice, these changes may play a role in promoting disinhibited exploratory behaviours in the EPM and OFT. Previous research has found intraperitoneal injections of a D_2_ antagonist to increase open arms entries and number of head dips in the EPM (Rodgers et al., 1994). Similarly, DAT knockdown in the NAc of mice increases time spent and number of entries in the open arms of the maze (Bahi and Dreyer, 2019). Altered ability to inhibit exploratory responses post-GCI may thus be influenced by diminished DRD_2_-ir in the NAcS and reduced DAT expression in the NAc observed in this study. Other biochemical signals may contribute to impulsivity and exploratory behaviour. For instance, stimulation of 5-HT_1B_ receptors and administration of selective serotonin reuptake inhibitors fluoxetine and paroxetine are known to reduce EPM exploration (Lin and Parsons, 2002; Drapier et al., 2007). More specific to ischemia, corticotropin-releasing hormone knockdown restricted to the hypothalamus led to decreased plasma CORT concentration and more EPM, OFT, and light-dark box exploration (Zhang et al., 2017), while the opposite has been noted upon CRH infusion into the NAcS prior to EPM and OFT (Chen et al., 2012).

The tendency for ischemic rats to show reduced ΔFosB signal intensity in the vmPFC could be an indicator of reduced neuronal activation (Vialou et al., 2015; Kormos et al., 2016). In this context, our observations present an interesting parallel with that of Feja and Koch (2014), in which muscimol inactivation of the rat vmPFC resulted in impaired impulse control, as assessed in the 5-Choice Serial Reaction Time Task (5-CSRTT), while it failed to affect lever preference in a DD task. Other groups have supported that lesions to the vmPFC alter inhibitory measures of control (Chudasama et al., 2003). This leads us to hypothesise that reduced vmPFC activation might contribute to the possible behavioural impulsivity in our ischemic rodents without impacting impulsive choice. Furthermore, the interaction between the hippocampus and the vmPFC could be essential in modulating response inhibition, as the separation of these two structures also results in a dysregulation of impulse control in the 5-CSRTT (Chudasama et al., 2012). The extensive CA1 hippocampal cell loss observed in ischemic rodents, combined with the diminished neuronal activity in the vmPFC, could help explain the behavioural disinhibition/impulsivity that seems to be exhibited by GCI rats. It is important to note that the EPM is classically known to measure anxiety-like behaviours, and that the interpretation of behavioural disinhibition in ischemic rodents remains novel. As such, the use of other measures and behavioural tests, such as the 5-CSRTT or the newly designed Elevated Gradient of Aversion (Rico et al., 2019), could allow better elucidation of the subtypes of impulsivity impacted by GCI.

## Conclusion

Our study is the first to demonstrate that global cerebral ischemia does not impact impulsive choice in a delay discounting task, irrespective of alterations to behavioural disinhibition witnessed in the Elevated-Plus Maze and significant reductions of dopaminergic and ΔFosB signalling. Furthermore, mesocorticolimbic expression of dopamine receptor D_2_, dopamine transporters, and ΔFosB support distinctive roles of the nucleus accumbens core and shell, and the ventromedial prefrontal cortex in regulating impulsive behaviour and impulsive choice in ischemic rodents. These novel findings highlight the selective nature of the behavioural impairments caused by global cerebral ischemia on specific subtypes of impulsivity and emphasise the need for future studies to further characterise post-ischemic behavioural disinhibition.

## Data Availability Statement

The raw data supporting the conclusions of this article will be made available by the authors upon reasonable request.

## Ethics Statement

All conducted procedures were in accordance with the Canadian Council on Animal Care and ARRIVE guidelines and were approved by the University of Ottawa Animal Care Committee (protocol number PY 3278).

## Author Contributions

AM contributed to conceptualisation, methodology, validation, formal analysis, investigation, data curation, supervision, project administration, writing – original draft, review, and editing. MP contributed to validation, formal analysis, investigation, data curation, writing – original draft, review, and editing. HP contributed to conceptualisation, validation, funding acquisition, writing – original draft, review, and editing, supervision. All authors contributed to the article and approved the submitted version.

## Conflict of Interest

The authors declare that the research was conducted in the absence of any commercial or financial relationships that could be construed as a potential conflict of interest.

## Publisher’s Note

All claims expressed in this article are solely those of the authors and do not necessarily represent those of their affiliated organizations, or those of the publisher, the editors and the reviewers. Any product that may be evaluated in this article, or claim that may be made by its manufacturer, is not guaranteed or endorsed by the publisher.
